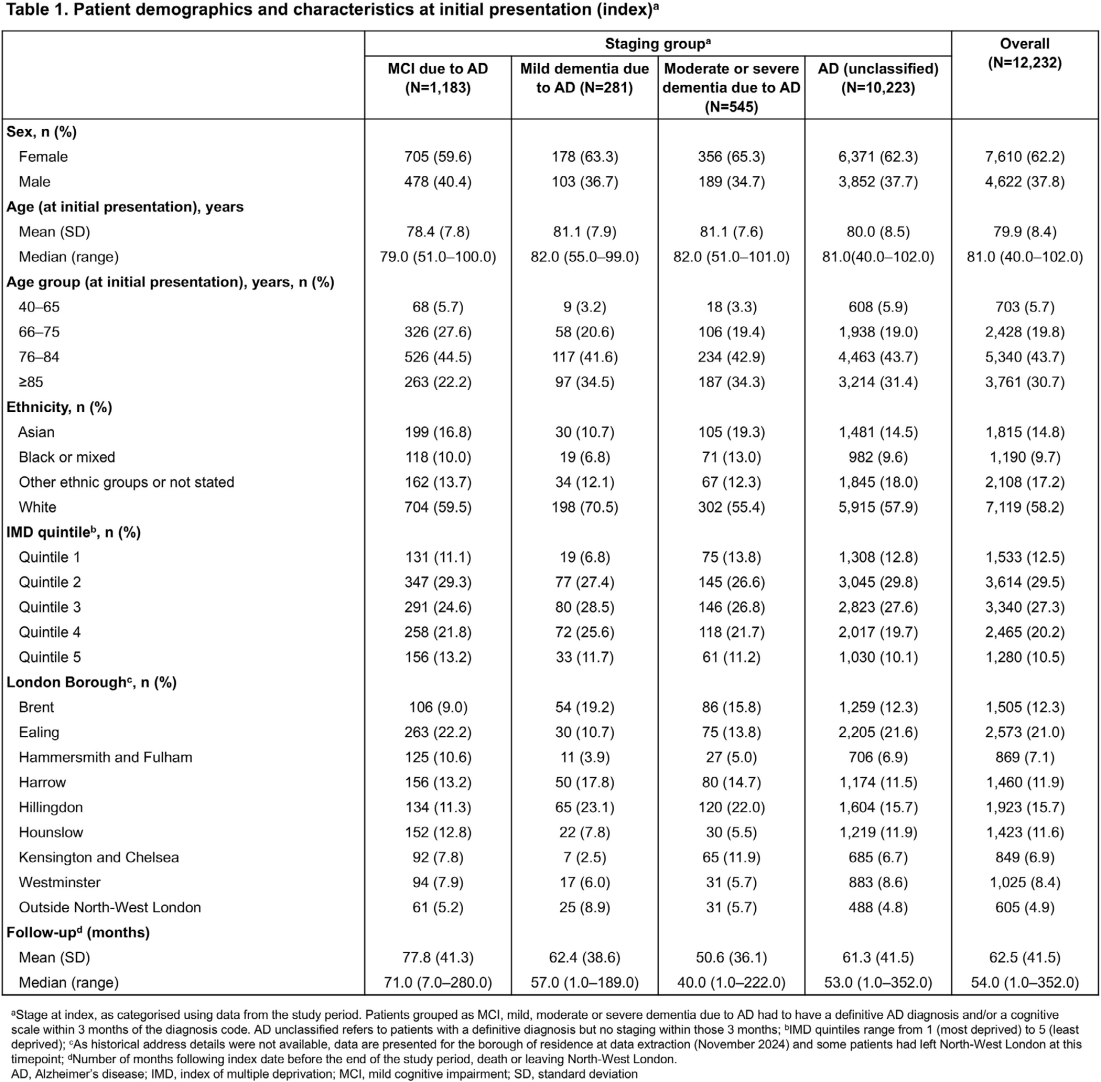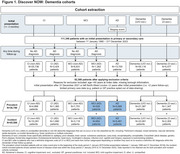# Characteristics and staging of patients with Alzheimer's disease: real‐world data from the United Kingdom

**DOI:** 10.1002/alz70860_099035

**Published:** 2025-12-23

**Authors:** Caroline S Casey, Heather E Fitzke, Ben S Rothwell, Alice S Farquharson, Niraj S Patel, Lill‐Brith von Arx, Marcus Yarwood, Joanne E Findlay, Ruth Mizoguchi

**Affiliations:** ^1^ Eli Lilly and Company, Basingstoke, Hampshire, United Kingdom; ^2^ Imperial College Health Partners, London, United Kingdom; ^3^ Eli Lilly Danmark A/S, Herlev, Copenhagen, Denmark; ^4^ Chelsea and Westminster Hospital NHS Foundation Trust, London, United Kingdom

## Abstract

**Background:**

Alzheimer's disease (AD) prevalence is increasing, with high burden felt by patients, families and healthcare systems. Early and accurate diagnosis of AD, including dementia severity, is important to ensure patients receive appropriate support, and treatment where available. The status of real‐world reporting of AD patient characteristics and disease staging, and how accurately it reflects the clinic, is unclear.

**Methods:**

This retrospective cohort study used electronic health records from the Discover NOW secure data environment. Characteristics and staging data were extracted for patients aged ≥40 years registered at a primary care general practice in North‐West London between 2015–2023, with onset of cognitive impairment, mild cognitive impairment (MCI) or AD (Figure 1). Possible AD staging events included global deterioration scale, cognitive testing, diagnoses or care plans indicative of disease severity.

**Results:**

Of 12,232 patients with MCI or dementia due to AD, 62.2% were female, mean age at initial presentation was 79.9 years and 58.2% were white (Table 1). At index 1,183 had MCI due to AD, 281 had mild AD dementia and 545 had moderate or severe AD dementia. Most patients (10,223) had no classified disease stage recorded within 3 months of diagnosis and 6,887 patients (67.4%) with unclassified AD received no staging events (19.1% received one event, 13.6% received ≥2 events) during the study. Most patients with staged AD (*n* = 826; mild/moderate/severe dementia) received one (51.0%) or two (24.5%) staging events (24.6% received ≥3 events). In the overall cohort, 16.5% of patients presenting with MCI (*n* = 988/6,000) received a subsequent AD diagnosis within 5 years and 19.7% (*n* = 1,183/6,000) received an AD diagnosis at any time point.

**Conclusion:**

These data indicate a lack of recorded staging events for patients with AD: fewer than 1 in 10 patients diagnosed with AD received a classified disease stage recorded within 3 months of diagnosis. Most received no staging events throughout the entire 8‐year study period. This highlights both the low AD staging rate in current UK practice and limitations of available real‐world data on staging and disease progression. Further efforts are needed to ensure timely staging, enabling patients to access appropriate treatment and support.